# General considerations on posterior fossa arteriovenous malformations (clinics, imaging and therapy)

**Published:** 2010-02-25

**Authors:** A Neacsu, AV Ciurea

**Affiliations:** 1st Neurosurgical Clinic, ‘Bagdasar–Arseni’ Clinical Hospital, BucharestRomania

**Keywords:** arteriovenous malformation, infratentorial location, posterior fossa, multimodality treatment

## Abstract

The posterior fossa arteriovenous malformation (AVM) is uncommon and different from other intracranial AVM in its natural history, diagnosis, treatment, prognosis, and other features. Authors present a review of the actual procedures of diagnosis and treatment in the field of infratentorial cerebral arterio–venous malformations, based on the actual literature data. Pre–therapeutic considerations, such as clinical presentation and diagnostic evaluation, are initially discussed because they are crucial in choosing the optimal treatment. Posterior fossa AVMs merit multimodality intervention when feasible in most cases because of their higher risk of rupture and higher potential of morbidity and mortality. In addition, we present two significant cases treated in our department.

## Introduction

Clingenstein [43] first reported Infratentorial arteriovenous malformations as a clinical entity in 1908, and, Olivecrona and Rives have reported the first successful resection of a posterior fossa AVM in 1932 [24].

Although relatively rare lesions, infratentorial AVMs caused a great interest within the neurosurgical community. Significant efforts have been made for a better understanding of anatomic and hemodynamic complexity of these lesions, to facilitate more effective treatment strategies. 

Despite the marked emergence of microsurgical techniques, which have become more refined in the last half century, excision of posterior fossa AVMs is a formidable quest. The difficulty of treatment of these lesions, in terms of surgery, lies in the need for preservation of critical neurovascular structures within or around the brainstem and cerebrum, and the need for preservation of cranial nerve and deep brainstem nuclei, plus technical difficulties caused by the narrow surgical corridor, especially for deep lesions [28, 31,35]. Accumulating experience with the treatment of posterior fossa AVMs and the incorporation of multimodality approaches, including radiosurgery, endovascular therapy, and improved microsurgical techniques, have significantly contributed to continuously improving outcomes [23,34].

Success of treatment mainly depends on advanced medical technology, namely: digital subtraction angiography, MRI angiography, neuroradiologic interventional procedures, intraoperative monitoring, neuronavigation, neuroanesthesia, with controlled hypothermia, the use of neurosurgical microinstruments, and the existence of a radiosurgical department.  

## Diagnosis and clinical manifestations

Natural History. Posterior fossa AVMs account for only 5–7% of all intracranial AVMs. With the advances of modern neuroimaging, the frequency rate of posterior fossa AVMs grew to 10–15% [1,2,6,7]. The incidence of posterior fossa AVMs at autopsy is even higher, reaching 20% of all intracranial AVMs.

Infratentorial AVMs may be located within cerebellum, brainstem or both. McCormick [19] has published a study of 104 cases of infratentorial AVMs, in which 69 were located within the brainstem, most commonly in the ponts. Batjer and Samson [2] showed that two thirds of the posterior fossa AVMs are located within the cerebellum, 20% in the brainstem and 20% in both cerebellum and brainstem.

Bleeding is the most common form of presentation of posterior fossa AVMs [3,10, 21,38]. In most clinical series the incidence of subarachnoid or intraparenchimatal bleeding was reported to be between 75 and 92% [13,25,26]. Recent studies have suggested that brainstem AVMs presentation is less common with bleeding than cerebellum AVMs. Accumulating data have demonstrated an independent association of infratentorial AVM location and hemorrhagic presentation [13,26, 41]. This is alarming in light of the considerably greater morbidity and mortality associated with posterior fossa AVM rupture [12]. Fortunately, with accumulating surgical experience and the cultivation of multimodality AVM therapy, therapeutic success continues to improve [25]. Posterior fossa AVMs were frequently associated with aneurysms (25%) on the feeding arterial pedicles of the nidus and were often the cause of hemorrhage [20,22, 39].

Progressive neurological deficits (including those secondary to mass effect, ischemia, and hydrocephalus) were the second most common mode of presentation [37]. Cranial nerve palsies, often affecting the trigeminal nerve, have been associated with lesions of the cerebellopontine angle and brain stem [6]. Headache, a relatively non–specific symptom, can occur in as many as 10% of the patients diagnosed with unruptured AVMs [2]. At the time of detection, at least 15% of people affected by AVMs are asymptomatic [30]. The incidence of asymptomatic posterior fossa AVMs may rise in the future with the increasing use of advanced neuroimaging modalities for nonspecific symptomatology [25]

Complete **neuroradiological evaluation** of AVMs includes cerebral computer tomography (CT) with contrast administration, magnetic resonance imaging (MRI) and angio–MRI, cerebral angiography with substraction.

Because the posterior fossa AVMs present with acute symptoms, the initial diagnostic test is usually **computer tomography** (CT) scan. Brain CT–scan is a first diagnostic neuroimaging of either the AVM itself, of intraparenchimatal hematoma secondary to malformation rupture, or subarachnoid or intraventricular bleeding. Brain CT–scan, with or without contrast is the screening procedure of unruptured posterior fossa AVMs: grouped serpentine veins and dilated arteries, and, sometimes, large drainage veins may be revealed, elements that highly suggest the diagnosis of AVM. CT–scan may provide information regarding location, extent and size of the AVM, but the value of CT–scan is limited regarding diagnosis and anatomical evaluation of AVM, when compared to angiography and magnetic resonance imaging.

**Magnetic resonance imaging** (MRI) combined with angio–MRI allows specific diagnosis in most cases with AVMs. Information provided by MRI exam supplement those provided by angiography, improving the tridimensional definition of AVM. 

Angio–MRI allows multiplanar evaluation of the AVM, but does not identify all feeding arteries and drainage veins of the complex AVMs [33]. In order to identify feeding arteries, selective arterial angio–MRI for a single arterial branch can be used to evaluate drainage veins MRI venography. Moreover, sensory ‘phase contrast ’ technique may be associated and can be used to reveal slow flow. Angio–IRM is useful (together with conventional MRI, which more accurately reveals the AVM topography) in planning surgical treatment, embolization or irradiation with gamma–knife of the AVM, however, it is insufficient. 

Posterior fossa AVM functional MRI [15], helps to a more precise delineation of target volume for radiosurgery (although it is more useful in supratentorial lesions compared to subtentorial ones). Functional MRI also allows study of brain functions reorganization and postoperative recovery. 

Gold–standard diagnosis is represented by angiography, and if necessary, stereo–angiography. It is the most useful and sensitive method to identify and evaluate the AVMs (provides the richest information regarding nidus characteristics, feeding and drainage vessels), and to highlight the operatory planning of the AVM. All patients must undergo formal six–vessel catheter angiography for accurate characterization of the anatomy and hemodynamics of the AVM. In particular, all feeding arteries and draining veins must be diligently identified preoperatively, in the preparation for a complete resection. High– resolution magnification studies are required for both VAs, both internal carotid arteries, and both external carotid arteries, because approximately 10% of infratentorial AVMs are fed by one or both external carotid arteries [27, 31,32]. No other investigation cannot replace conventional DSA angiography in therapeutic decision. 

Optionally, exploration of cerebral hemodynamics can be done by: positron emission tomography (PET) or SPECT. 

Intraoperative ultrasonography is useful to highlight any remaining portion of the AVM, but also to test the functionality of the remaining blood circulation after cliping. 

## Treatment options

The main goal of treatment is complete cure of the lesion and prevents the risk of bleeding with restoring normal brain flow. It is considered that the complete absence of AVM on postoperative angiography eliminates the risk of secondary hemorrhage.

The choice of treatment for patients should consider risks attendant to each therapeutic option, as well as the natural history of the individual patient [18]. Current treatment methods include radiosurgery, open surgery, endovascular embolization and combination of them. Occasionally, patients with asymptomatic AVMs may not be treated but should be followed clinically on an annual or semiannual basis [34]. Treatment should be individualized for each patient and should be undergone by a multidisciplinary team.

Most neurosurgeons prefer to leave the small, unruptured malformations, located in eloquent areas or in elderly patients, for radiosurgery. AVMs under 3 cm in diameter, located in non– eloquent areas are fit for surgery. Small, ruptured AVMs, located in eloquent areas, which have caused neurological deficits, are treated with open surgery.

Surgery for medium and large AVMs depends on a series of objective factors mentioned above but also on subjective factors (department's tradition, neurosurgeon's experience, family desire, etc.)

Ruptured AVMs with hematomas, prone to causing brain herniation, require emergent surgery, as a first step, in order to evacuate the hematoma, the angiography and the curative surgery are done later.

Patients with major deficits, which occurred after rupture of malformations, but without imminent herniation can be treated conservatively, until neurological stabilizing, followed by reevaluation and surgery.

Patients with large, unruptured, oligosymptomatic AVMs, located in eloquent areas, carrying particularly high–risk for surgery are treated conservatively and followed–up.

With the sole exception of very small AVMs, embolization is beneficial before surgical treatment of most AVMs in the posterior fossa.

Each of the above mentioned methods have several limitations:

1. surgery cannot resolve deep anatomical lesions or patients presenting with altered neurological status.
2. radiosurgery has a high rate of complications in AVMs larger than 3cm.
3. embolization is not a radical cure, except for 5–10% of cases, but it can proceed in an open surgery in giant AVMs, or radiosurgery; it can treat AVM–associated aneurysms and arteriovenous fistulae from plexiform malformations.

Analysis of late results of different treatment combinations is demanding and requires long–term angiography: embolization carries the risk of revascularization and radiosurgery has a delayed action.

### Pre–therapeutic considerations

The next three steps must be rigorously taken when evaluating each case:

1. Comparisons between treatment risks and risk required by the natural history of disease
2. Evaluation of the patients' age, comorbidities, pregnancy in women, assessing the location of the lesion with extensive anatomical details including venous drainage, which are the guiding factors for therapy selection.
3. Comparison of risk factors between different procedures or combinations of procedures.

Therapeutic decision requires a thorough knowledge of the risks and benefits of different options, in such a way that the patient receives the best choice.

Choosing the optimal treatment depends on the clinical condition, size, location and angio–architecture of the AVM, patient's age, comorbidities, and treatment possibilities of the team. The treatment method chosen must have a lower risk than the natural history of the disease, involving a bleeding rate of 6–15% per year for posterior fossa AVMs [3,8,9,11,42]. 

### Grading Systems and Risk of Therapy

Angiography allows assessment of AVM's angio–architecture and hemodynamic situation entirely. Even medium AVMs (3–6 cm) can have multiple compartments requiring supraselective angiography necessary to adopt optimal intraoperative strategy.

It is important to specify: the presence of high flow, plexiform components, multiple feeding arteries, presence of lenticulostriat feeding arteries, which must be preserved during surgery, presence of aneurysms located into the nidus or on feeding arteries, venous drainage, which can sometimes flow extensively through the skull, endangering the craniotomy.

Martin–Spetzler scale [36] was adopted in 1986 and it is a widely used therapeutical decision–making tool which gives information about the risk assessment of postoperative neurological deficit in AMVs. [Table 1]

**Table 1:** Martin–Spetzler scale

Size of AVM	Points assigned
< 3 cm – small AVM	1
3–6 cm – medium AVM	2
> 6 cm – large AVM	3
Eloquent adjacent brain	
Noneloquent	0
Eloquent	1
Venous drainage	
Superficial	0
Profound	1

The grading of AVM is given by the sum of points assigned for size, eloquence and venous drainage.

The higher the malformation, the higher the intraoperative risks are, hyperperfusion syndromes occur more frequently and the risk of postoperative deterioration becomes more serious.

The risk of postoperative neurological deficits for small lesions is 5.1%, for medium AVMs is 21.5% and for large ones it reaches 40%. Deep venous drainage, adds a plus of 20% in permanent neurological deficits.

It is generally considered that patients with AVMs of grades Ⅰ, Ⅱ and Ⅲ benefit from surgical resection and those with grades Ⅳ and Ⅴ have major risk of permanent neurological deficits. Grade Ⅵ patients are considered inoperable.

### Surgical Treatment

Arteriovenous malformations of the infratentorial space pose a unique technical challenge for neurovascular surgeons. Until recently, the surgical risk of resection of arteriovenous malformations in this location was considered unacceptable. However, despite the increasing experience and the refinements in surgical and anesthetic techniques, procedures once considered to court disaster can now be achieved with acceptable morbidity rates [4,21]. Successful removal of posterior fossa AVMs requires an intimate knowledge of the architecture of the malformations, the surrounding anatomical structures, and their involved relationships [4].

Because most posterior fossa AVMs present with intraparenchymal hemorrhage, the initial surgical focus is directed to hematoma removal and treatment of any acute mass effect or hydrocephalus, if it is present. When feasible, however, definitive resection of the AVM should be deferred for 4 to 6 weeks. In this manner, delayed extirpation often ensures sufficient time for brain swelling to diminish and the hematoma to liquefy, features that greatly facilitate ultimate AVM excision. Late treatment is also beneficial in that associated cerebral aneurysms, which might initially have been obscured by intraparenchymal hemorrhage on the first angiogram, and could later be clearly visualized after a brief period of quiescent. On rare occasions, a patient who needs early hematoma evacuation may require complete AVM resection during the same operation because of intraoperative hemorrhage. In such a situation, every effort is made to preserve all viable cerebellar tissue as well as alleviate any undue mass effect on the brainstem.

Basic principles for surgical treatment of posterior fossa AVMs are similar to those for treatment of AVMs with any other intracranial location [27]. The principles of optimal patients positioning, adequate bony exposure, extensive dural opening, meticulous attention to sharp microdissection, and compulsive hemostasis are critically important during surgical resection of posterior fossa AVMs [4,16].

**Fig 1:**
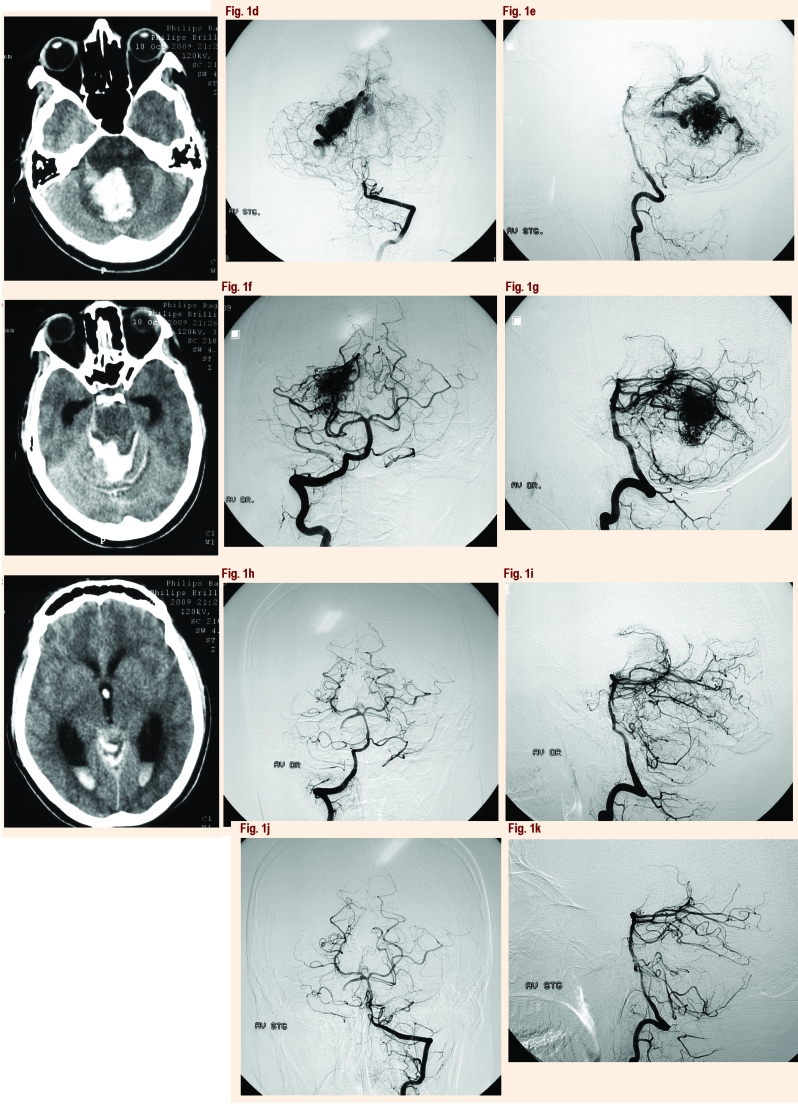
A 19-year-old woman admitted in comatose state found to have cerebellar AVM: (a),(b),(c) axial CTscan demonstrating vermian hemorrhage with important surrounding edema, intraventricular bleeding within the third and forth ventricle, massive infratentorial subarachnoid hemorrhage and acute hydrocephalus;(d),(e) left vertebral angiogram and (f),(g) right vertebral angiogram showed an infratentorial AVM, located within the right cerebellar hemisphere, with a nidus, measuring around 3 cm – the maximal diameter in the craniocaudal direction with arterial feeders from the right posterior cerebral artery and superior cerebellar artery and venous drainage into the Galen vein, Herophil torculla, and right lateral sinus; (h),(i),(j),(k) postoperative angiographic studies obtained in the same patient: anteroposterior and lateral projections reveal complete resection of the cerebellar AVM and excellent flow through the vertebrobasilar circulation.

There are neurosurgeons who prefer to operate all types of AVMs. Most neurosurgeons recommend surgery with resection of infratentorial AVMs, as the treatment of choice (excluding patients with comorbidities, that increased surgical risk of patients over 70 years old), due to the high morbidity associated with bleeding in the posterior fossa [34,38]. With the help of modern microsurgical instruments, the modern means of neuroprotective neuroanesthesia (heart stopping and extracorporeal circulation, deep hypothermia with the central body temperature of 15 degrees Celsius for a maximum period of time of 50 minutes of work), MAV located in cerebellum, subarachnoid cisterns, and pial surfaces of the brainstem may be successfully treated surgically with excellent results in most cases.[27,34].However, in certain situations, resection carries an unacceptable risk of injury to the patient. Contraindications of surgical removal include brainstem AVMs that do not present to the pial surface, cerebellar AVMs that involve the deep cerebellar nuclei, poor neurological or medical condition of the patient, and advanced age. [34][Fig 1]

### Endovascular Treatment

The goal of embolization is to permanently reduce the size of malformations and abnormal blood flow thereby, reducing the incidence of secondary bleeding, and favoring other curative procedures, surgical or radiosurgical, with minimum risks for the patient. 

Current indications for embolization can be divided into presurgical embolization in large or giant cortical AVMs and embolization before radiosurgical intervention to reduce nidus size. In addition, palliative embolization may be used in large nonsurgical or nonradiosurgical AVMs in patients presenting with progressive neurological deficit secondary to high flow or venous hypertension.

Many posterior fossa AVMs can be treated with preoperative endovascular embolization to decrease the size and morphology of the AVM. Embolization is often completed in several stages to minimize the risk of breakthrough bleeding that can occur when a large volume of an AVM is embolized in a single session [34].

Except for very small AVMs, embolization before surgery is beneficial in the vast majority of posterior fossa AVMs. Technical progress in endovascular therapy allows relatively easy catheterization of feeding arteries, although the widespread use of aggressive embolization increased general mobidity [5,29]. In a series of 150 patients with embolization of intracranial AVMs, Wikholm et al. reported a mortality rate of 1.3% and a rate of severe and moderate complication of 6.7% and 15.3%, respectively [40].

Because embolization is an adjunct to surgical resection of the lesion, embolization should be primary focused on various large feeding arteries or arteries difficult to be found early during surgery. In large cerebellar AVMs the feeding arteries come from PICA. Preoperative embolization of these arteries was shown to lower blood flow through the nidus. Because PICA is relatively quickly exposed during surgery, proximal embolization of PICA is not needed, because of the high risk of cerebellum or brainstem stoke.

Feeding arteries from ventricular branches of AICA are difficult to expose during surgery in any posterior fossa approach, therefore preoperative embolization of these arteries is helpful.

Embolization of feeding arteries from SCA may be indicated if SCA is dilated by increasing blood flow and it is the source of significant feeding arteries. If feeding arteries from SCA are small, it is difficult or impossible to selectively catheterize these vessels. In these conditions, proximal embolization of SCA should be avoided because of the risk of cerebellar stroke. Feeding arteries from distal SCA can be addressed surgically adjacent to the nidus during surgical resection of the AVM. Venous drainage of the AVM in this location usually goes to the galenic system through precentral cerebellar veins, and preoperative embolization of branches from SCA may facilitate surgical dissection of the tentorial part of the cerebellum.

Preoperative embolization has no significant utility in the treatment of brainstem AVMs due to the risk of brainstem stroke.

### Radiosurgery

Stereotactic radiosurgery has become an important treatment technique for the management of cerebral AVMs. The purpose of radiosurgery is to irradiate the blood vessels of the AVM to cause progressive luminal obliteration and thereby prevent hemorrhage.

Traditionally, radiosurgery is indicated for AVMs located in eloquent areas associated with high surgical morbidity, such as the basal nuclei and brainstem. The goal of radiosurgery obliteration of AVMs with nidus volume between 5 and 10 cubic cm. Complete obliteration is done in a latency of 1–3 years after radiosurgery in 80–85% of cases, while the patient is not protected from bleeding. 

**Fig 2:**
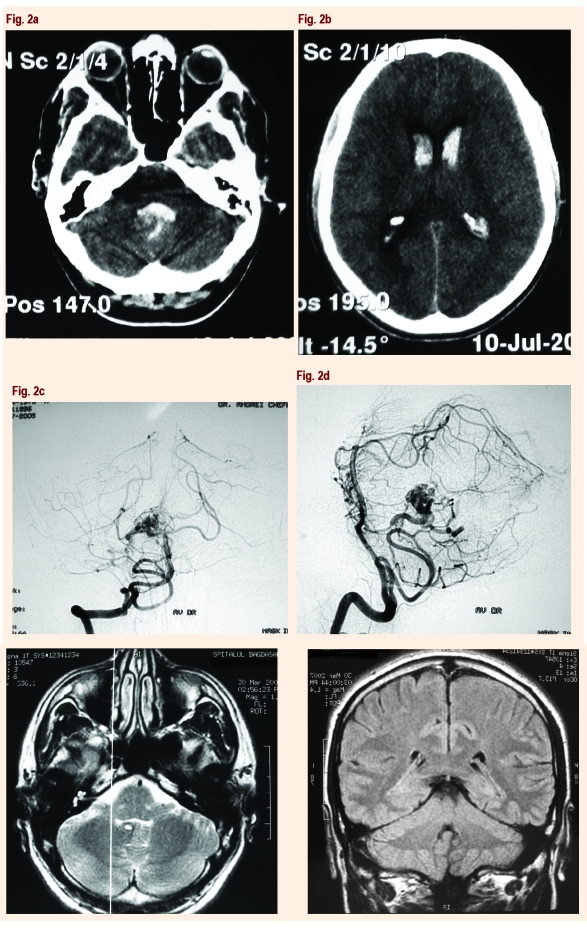
Pretreatment imaging studies of a 30–year–old man with basal panventricular hemorrhage as seen in axial CT in (a),(b); (c) anteroposterior and (d) lateral right vertebral angiogram showed a cerebellar AVM, located within the right cerebellar hemisphere, with a nidus, measuring around 1,5 cm maximal diameter, with arterial feeders from the right anteroinferior cerebral artery and venous drainage into both lateral sinus and straight sinus; axial (e) and coronal (f) MRI scan show a small vermian, right paramedian, arteriovenous malformation  with minimal mass effect.

**Fig 3:**
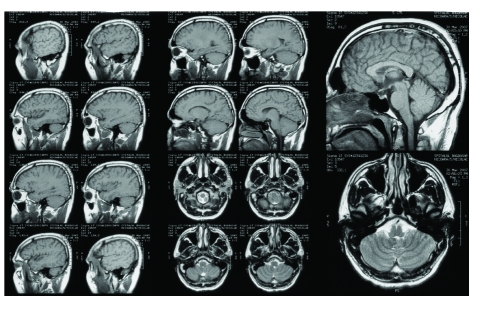
MRI studies (axial, coronal and sagital scans) obtained in the same patient two years post gamma-knife treatment showing almost completely obliteration of AVM.

**Fig 4:**
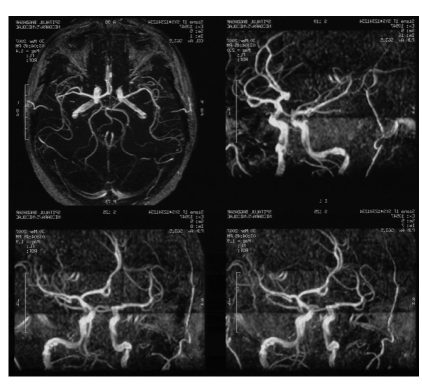
Angio-MRI two years post gamma-knife treatment.

Massager et al [17] studied the results of 87 patients with brainstem AVMs treated with gamma knife and showed that 95% of patients have improved or remained neurologically stable, with an obliteration AVM rate of 63% in two years and 73% in three years. These results lead to the following guidelines: if the surgeon is experienced in AVM surgery, microsurgery is an excellent option for superficial, pial AVMs of the brainstem, while radiosurgery is the best treatment for deep brainstem malformations.[Fig 2, Fig 3, Fig 4]

### Multimodality Treatment of posterior fossa AVMs

AVMs are often treated by more than one treatment modality. This occurs in one of two fashions. It is done as either a planned maneuver, typically with embolization followed by surgical resection or radiosurgery, or as an unplanned maneuver where one treatment modality fails and a second treatment modality is necessary to obliterate the AVM. This can occur in situations such as residual AVM after subtotal surgical resection or resection of an AVM after incomplete radiosurgical treatment [14,23].

Endovascular embolization can be performed before surgical excision to reduce the difficulty of surgery, or before radiosurgery to bring the size of the lesion to the limits of the machine. 

Radiosurgery may be used to eradicate small residual disease left after craniotomy (due to technical difficulty or involvement of eloquent structures).

Multidisciplinary teams will probably become increasingly important for optimal management of each individual patient. Such teams may include neurosurgeons, interventional neuroradiologists, and stereotactic radiation specialists. Continuing advances in microsurgical, neurointerventional, and radiosurgical techniques will affect treatment approaches. 

## Conclusion

Posterior fossa AVMs deserve multimodality intervention when feasible in most cases because of their higher risk of rupture and higher potential for morbidity and mortality. Surgical resection remains the gold standard of therapy for treating symptomatic posterior fossa AVMs because it has the advantage of immediately removing the risk of hemorrhage in comparison with other treatment modalities. Although modern surgical techniques allow removal of cerebellar and superficial brainstem AVMs with low rates of morbidity and mortality, AVMs located primarily in the brainstem parenchyma are currently best treated with radiosurgery. Except for very small AVMs, embolization before surgery is beneficial in the vast majority of posterior fossa AVMs.
